# A randomized controlled trial of an Internet-based intervention for eating disorders and the added value of expert-patient support: study protocol

**DOI:** 10.1186/s13063-019-3574-2

**Published:** 2019-08-16

**Authors:** Pieter J. Rohrbach, Alexandra E. Dingemans, Philip Spinhoven, Elske Van den Akker-Van Marle, Joost R. Van Ginkel, Marjolein Fokkema, Markus Moessner, Stephanie Bauer, Eric F. Van Furth

**Affiliations:** 1 GGZ Rivierduinen Eetstoornissen Ursula, Postbox 405, Sandifortdreef 19, 2300 AK Leiden, the Netherlands; 2 Institute of Psychology, Leiden University, Leiden, the Netherlands; 3 Department of Psychiatry, Leiden University Medical Center, Leiden, the Netherlands; 4 Department of Biomedical Data Sciences, Section Medical Decision Making, Leiden University Medical Center, Leiden, the Netherlands; 5 Center for Psychotherapy Research, University of Heidelberg, Heidelberg, Germany

**Keywords:** Eating disorders, Internet, Internet-based, E-mental health, Intervention, Treatment, Prevention, Expert patient, Peer support, Cost-effectiveness

## Abstract

**Background:**

E-mental health has become increasingly popular in interventions for individuals with eating disorders (EDs). It has the potential to offer low-threshold interventions and guide individuals to the needed care more promptly. Featback is such an Internet-based intervention and consists of psychoeducation and a fully automated monitoring and feedback system. Preliminary findings suggest Featback to be (cost-)effective in reducing ED symptomatology. Additionally, e-mail or chat support by a psychologist did not enhance the effectiveness of Featback. Support by an expert patient (someone with a lived experience of an ED) might be more effective, since that person can effectively model healthy behavior and enhance self-efficacy in individuals struggling with an ED. The present study aims to replicate and build on earlier findings by further investigating the (cost-)effectiveness of Featback and the added value of expert-patient support.

**Methods:**

The study will be a randomized controlled trial with a two-by-two factorial design with repeated measures. The four conditions will be (1) Featback, in which participants receive automated feedback on a short monitoring questionnaire weekly, (2) Featback with weekly e-mail or chat support from an expert patient, (3) weekly support from an expert patient, and (4) a waiting list. Participants who are 16 years or older and have at least mild self-reported ED symptoms receive a baseline measure. Subsequently, they are randomized to one of the four conditions for 8 weeks. Participants will be assessed again post-intervention and at 3, 6, 9, and 12 months follow-up. The primary outcome measure will be ED psychopathology. Secondary outcome measures are experienced social support, self-efficacy, symptoms of anxiety and depression, user satisfaction, intervention usage, and help-seeking attitudes and behaviors.

**Discussion:**

The current study is the first to investigate e-mental health in combination with expert-patient support for EDs and will add to the optimization of the delivery of Internet-based interventions and expert-patient support.

**Trial registration:**

Netherlands Trial Register, NTR7065. Registered on 7 June 2018.

**Electronic supplementary material:**

The online version of this article (10.1186/s13063-019-3574-2) contains supplementary material, which is available to authorized users.

## Background

### E-mental health

Comorbidity, relapse, chronicity, and mortality are common in eating disorders (EDs), which indicates the seriousness of these psychiatric disorders [1, 2]. Unfortunately, many individuals with EDs do not receive appropriate healthcare [3]. A study in the Netherlands showed that it often takes many years to recognize that one is suffering from an ED and more than 4 years to seek treatment [4]. Explanations for not receiving fitting care seem to range from geographical and financial reasons to fear of loss of control, fear of stigmatization, and feelings of shame [5–7]. A simulation study by Moessner and Bauer [8] suggested that we can most effectively help people with an ED, not by improving existing treatments or aftercare, but by guiding them to care more quickly and focusing on prevention. Additionally, the earlier patients with an ED receive proper treatment, the higher the chances are for full recovery [9]. Recently, e-mental health (i.e., offering care or treatment via technological means such as websites, teleconferences, and smartphone applications) has been proposed as a solution to bridge this treatment gap that exists for individuals with an ED. E-mental health has the potential to reduce barriers to seek help, since it can provide inexpensive, anonymous, and easily accessible interventions [9]. Consequently, such low-threshold interventions could help to improve early detection and intervention of ED problems and to promptly guide individuals to more intensive care if needed.

Nevertheless, research regarding the effects of e-mental health on ED pathology and help-seeking behavior is still scarce. Results of a recent meta-analytic review [10] demonstrated that Internet-based programs, of which most relied on cognitive behavioral principles, successfully decreased ED-related symptoms such as body dissatisfaction, symptoms of bulimia nervosa, shape and weight concerns, dietary restriction, and negative affect, and increased self-esteem and self-efficacy. Two examples of Internet prevention interventions that have been proven effective in randomized controlled trials (RCTs) are Student Bodies [11] and the Body Project [12]. Student Bodies is a cognitive-behavioral Internet-based program, including psychoeducation, self-monitoring journals, behavioral exercises, and weekly assignments, aimed at improving eating- and body-related issues in people at risk for developing an ED. The Body Project appeals to the same group, but it employs a dissonance-based approach, by letting users critique the thin body ideal in written, verbal, and behavioral exercises. The strength of such interventions is their ability to reach an underserved population. However, they should not be seen as a replacement for face-to-face treatment, but rather as an addition to the stepped-care treatment of EDs [9]. Additionally, confidence in results from RCTs regarding e-mental health for EDs (covering a wide range of interventions but excluding studies in which the therapist was the primary means of delivering the intervention) is generally low (often because of the high risk of bias and inconsistency and the indirectness of and imprecision in outcomes), so more solid research regarding the form and content of such interventions is needed [13].

Naturally, there are limitations to low-threshold Internet interventions aimed at prevention and early intervention of EDs in the extent to which they can respond to the personal situation of users, especially when compared with interventions in which intensive and direct contact with a professional is possible, such as blended care. Nevertheless, it is important for low-threshold Internet-based interventions not to employ a ”one-size-fits-all” approach. Indeed, not everyone profits from or prefers the same content of treatment, and the Internet is a highly suitable medium to convey interventions in a flexible and interactive way. The Internet-based program ”Featback” combines prevention and (early) intervention for individuals with ED symptoms. The program aims to make users aware of their eating-related and underlying problems. Users are encouraged to share their problems with their environment and, for more severe problems, to seek professional help. The program can be used anonymously, reducing the barrier to subscribe. It contains psychoeducation, a fully automated symptom monitoring and feedback system, and weekly chat or e-mail contact with a coach. A more detailed account of Featback is presented in the “Interventions” section of this article. Featback is based on ES[S]PRIT [14], a program originally developed in Germany. Research on ES[S]PRIT suggested the intervention is both feasible [14] and acceptable [15] and improves self-efficacy in young individuals with ED-related problems [16] and enhances help-seeking behaviors [17, 18].

The current research group has performed a first RCT investigating the effectiveness and cost-effectiveness of Featback [19]. Featback was offered with or without chat, Skype, or e-mail support from a therapist, which resulted in four conditions: (1) Featback only, (2) Featback with weekly support from a therapist, (3) Featback with support from a therapist three times a week, and (4) a waiting list control condition when Featback was complemented with therapist support once or three times a week. It was found that Featback (with or without support) was more effective in reducing symptoms of bulimia nervosa (*d* = − 0.16) and symptoms of anxiety and depression (*d* = − 0.31) than the waiting list control [20]. Contrary to our expectations, no difference in effectiveness between the active interventions was found. Regarding the cost-effectiveness, it was found that Featback with or without therapist support represented good value for the money when compared to a waiting list [21], indicating that Featback might be a good alternative to care as usual, especially for individuals who experience difficulties in seeking professional help. Although no added effect on ED psychopathology was found, Featback users were significantly more satisfied with the intervention when Featback was complemented with weekly or three-weekly therapist support. Finally, moderator analyses showed that Featback was most effective for individuals with mild to moderate bulimia nervosa symptoms [22]. The present study aims to follow up and build on these findings by further investigating the (cost-)effectiveness of Featback and by investigating the added value of expert-patient support.

### Expert-patient support

An explanation of why additional support from a psychologist did not add to the effectiveness of Featback [20] may be that although individuals suffering from ED symptoms appreciate the empathy and support of therapists, it may not be enough to reduce ED psychopathology. Support by expert patients (i.e., recovered individuals with a lived ED experience, also referred to as peers or mentors) may prove to be more effective for those reluctant to seek help and in the aftercare for individuals who have completed treatment and are at risk for relapse [23]. Specifically, expert patients may be more effective in changing behavior and inspiring hope of recovery, because of a perceived similarity and credibility [23]. The self-evident credibility of expert patients may make their interventions more valuable, since reliable [24] and personalized [25] messages are found to be more effective in changing behavior. Additionally, the shared experiences and accompanying (perceived) similarity enhances experienced social support and feelings of closeness [26–30] and various bonding behaviors [31, 32]. The idea that people who share a common background or problem have a unique resource to offer each other appears to be at the heart of peer support [33]. Relatedly, expert patients are thought to be effective in enhancing self-efficacy in patients, since they can powerfully model health behaviors and enhance patients’ belief in their own capabilities [23, 34, 35], which is one of the primary goals of Featback. Concordantly, in the current study it is hypothesized that the credibility and the shared background of an expert patient and participant are sufficient to establish feelings of closeness and make participants more receptive to interventions aimed at reducing ED symptomatology and enhancing experienced social support and self-efficacy.

The body of literature on the effectiveness of support by expert patients is growing. Many studies have investigated expert-patient support for patients with chronic somatic illnesses in comparison to treatment as usual and found positive, albeit small, effects on self-efficacy, self-management, illness-related quality of life, and worry about the illness [33, 36–41]. Adding expert-patient support to usual care has also been found cost-effective for patients with various chronic somatic illnesses [42]. However, results on the effectiveness of expert-patient support in mental illness are mixed. For example, in some studies expert-patient support was associated with reductions in depressive symptomatology in patients with major depression and was found to be as effective as professionally administered treatment and superior to a waiting list [43, 44]. Furthermore, increases in self-efficacy were found in patients with severe mental illness who received expert-patient support in addition to treatment as usual in comparison to patients who received treatment as usual only [45]. On the other hand, several studies found that adding expert-patient support to treatment as usual had no significant effect on psychopathology, quality of life, empowerment, or user satisfaction [46]. In addition, the type and objectives of the expert patient interventions are highly heterogeneous [47], and confidence in both positive and null findings is repeatedly low because of the high risk of bias [46]. This complicates assessment of the value of expert-patient support for mental illness and warrants further research.

Regarding EDs, currently only a few studies have been completed [48]. Perez, Kroon van Diest, and Cutts [49] report that individuals recovering from an ED who are assigned to an expert patient indicate better relationships, a higher quality of life, and increased intervention usage than recovering individuals who are not assigned to an expert patient. Results of two pilot studies are in line with these findings [50, 51]. However, there were no active control conditions in these studies. Additionally, Cardi et al. [52] and Beveridge et al. [53] are currently conducting trials on the topic of expert-patient support and EDs. In summary, expert patients may have positive effects on self-efficacy, belonging, and psychopathology [48], but findings are currently too circumstantial to provide convincing proof for the effectiveness of expert-patient support for EDs or recommendations on its implementation, so further investigation is necessary.

### Aims and research questions

The current study builds on the study by Aardoom et al. [20] by investigating whether the results regarding the effectiveness of Featback will hold. More specifically, the first aim is to investigate the (cost-)effectiveness of the Internet-based intervention Featback in comparison to Featback with support from an expert patient, support from an expert patient without Featback, and a waiting list control condition (WLC). The primary outcome measures of the current study are ED-related attitudes. Secondary outcome measures include self-efficacy, social support, symptoms of depression and anxiety, motivation to change, user satisfaction, intervention usage, and help-seeking attitudes and behaviors. Finally, cost-effectiveness will be evaluated through the reported quality of life, outcomes for patients in terms of capabilities, and medical and societal costs. We have two hypotheses accompanying the first research aim.

1. Our primary hypothesis is that Featback without expert-patient support, Featback with expert-patient support, and expert-patient support without Featback will be more effective in reducing ED psychopathology and more cost-effective compared to a waiting list.
2. Secondly, we hypothesize that the combination of expert-patient support plus an online intervention will be more effective in reducing ED psychopathology and more cost-effective compared to expert-patient support or Featback only and that the improved effectiveness will be maintained up until a year later.

The second aim of this study is to investigate predictors and moderators of intervention response to explore what works for whom. Predictors and moderators that will be tested as predictors or moderators of treatment response and/or intervention usage are age, gender, and educational level, motivation to change, social support, severity of ED symptoms, severity of symptoms of depression and anxiety, self-efficacy, self-esteem, and closeness or perceived similarity of participants with expert patients. Additionally, self-efficacy is examined as a mediator. Besides the exploratory tests, there are two hypotheses concerning the second aim of this study.

(H3) Since a perceived similarity of participants to expert patients might enhance the receptivity and self-efficacy of participants, it is hypothesized that participants who feel more similar to the expert patient they are assigned to have better outcomes in terms of ED symptomatology, self-efficacy, and experienced social support.
(H4) It is hypothesized that, since the effectiveness of expert patients is theorized to come from effectively improving self-efficacy, changes in self-efficacy during the intervention period mediate subsequent long-term effects of the intervention on ED-related symptoms and experienced social support.

Thirdly, we aim to investigate practical experiences with Featback, such as intervention usage and user satisfaction.

(H5) It is expected that Featback with weekly expert-patient support will enhance intervention usage as well as satisfaction with the intervention compared to Featback alone.

## Methods

### Design

Since the present study is a continuation of previous work of this research group, the methodology described here will be similar and in some parts identical to the previous design [19]. The current study describes an RCT with a two-by-two factorial design with repeated measures to create four different conditions: (1) Featback, comprising psychoeducation and a fully automated self-monitoring and feedback system, (2) Featback with weekly individualized support by an expert patient through e-mail or chat, (3) weekly individualized support by an expert patient through e-mail or chat, and (4) a waiting list. A description of the content of the interventions for each condition is presented below. After screening, all eligible participants are asked to give informed consent and fill in online baseline measures (T0). Subsequently, they will be randomized to one of the four conditions. An independent researcher will conduct randomized allocation by using the SPSS function to produce random numbers. Hence, the main researcher will be blind to the randomization process. Randomization will take place in blocks of 40 participants. The current design does not allow expert patients to be blinded to the study goal, since they are required to help participants with their ED or ED-related problems to the best of their abilities within the intervention protocol. Naturally, expert patients know that the individuals they have contact with via e-mail or chat are randomized to one of the expert-patient support conditions. Similarly, participants are not blinded concerning the condition allocation.

**Fig. 1:**
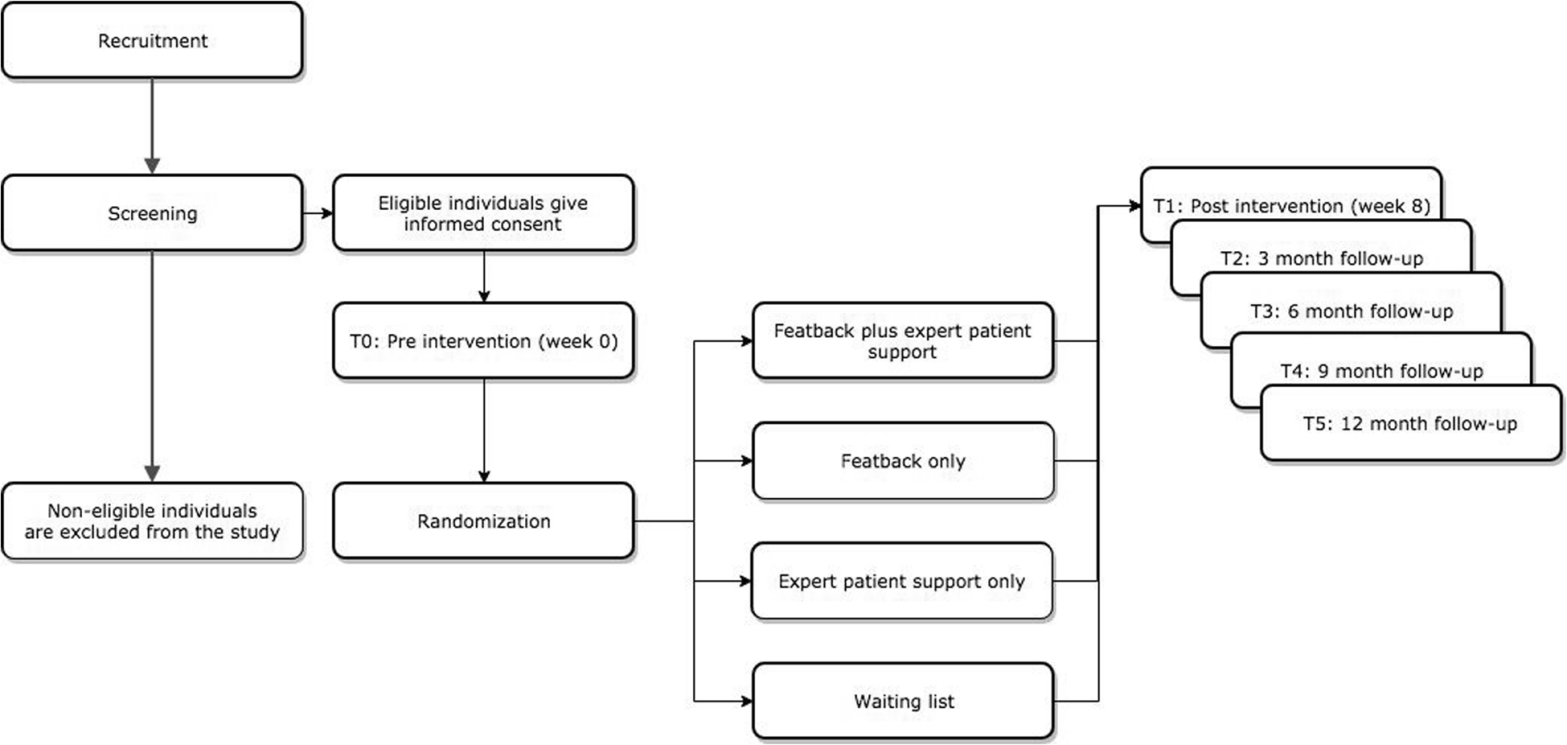
Flowchart of study procedures

After 8 weeks (intervention period or waiting period), participants are invited to complete the post-intervention assessment (T1). Finally, a link to the online follow-up questionnaires will be sent to them 3 (T2), 6 (T3), 9 (T4), and 12 (T5) months after T1 (see Fig. 1). Ethical approval has been obtained by an independent medical ethics committee (CME LUMC Leiden, file number NL64553.058.18). The Standard Protocol Items: Recommendations for Interventional Trials (SPIRIT) checklist is provided as Additional file 1.

### Participants

The study sample will be recruited via the Dutch e-community ”Proud2Bme” (http://www.proud2bme.nl), via the Featback website and the network of the patient organization WEET. Proud2Bme is an interactive website that is designed for young people or adolescents (mainly girls) with eating problems or an ED. It is a healthy alternative to many pro-anorexia websites and promotes a healthy lifestyle and positive self-image. Eligible participants are aged 16 years or older, have access to the Internet, have self-reported ED symptoms defined as scoring 52 or higher on the Weight Concerns Scale (WCS) [54], or report one or more of the following ED symptoms assessed by the Short Evaluation of Eating Disorders (SEED [55]): a body mass index (BMI) lower than or equal to 18.5, one or more binge eating episodes a week over the past 4 weeks, or one or more compensatory behaviors a week over the past 4 weeks. Participants are excluded if they are younger than 16 years or do not report any ED symptoms. Otherwise, there are no exclusion criteria, since both people with beginning and severe eating problems may benefit from Featback and/or expert-patient support (see also the “Ethical considerations” section of this article).

### Interventions

#### Featback

All participants in the Featback conditions can access the Featback website on which comprehensive information on EDs and their causes and consequences can be found (i.e., psychoeducation). The psychoeducation will be purely self-guided, meaning that participants are free to choose what to read and when. For the monitoring and feedback system, participants receive an invitation by e-mail to complete a monitoring assessment weekly. This questionnaire consists of four 4-point Likert items assessing ED-related behaviors, namely (1) excessive concerns with body weight and shape, (2) unbalanced nutrition and dieting, (3) binge eating, and (4) compensatory behaviors. When participants have completed the questionnaire, a supportive feedback message will be automatically generated according to a pre-defined algorithm, which addresses their reported behaviors (healthy or unhealthy range) and patterns of change (improved, deteriorated, or unchanged) on each of the assessed ED-related behaviors. Hence, the automated messages vary in content depending on the problems that users report. The messages contain a summary of self-reported eating problems, psychoeducation, and guidance on how to counter ED-related symptoms, which are formulated in a supportive and reinforcing way. Table 1 illustrates one of these automatically generated messages.

**Table 1:** Example of an automatically generated Featback message. *Note*: This message reflects the content when a participant indicates in the weekly monitoring that she or he worries about food, has at least one day in which she or he restricts food intake, and has had more binges and compensation behavior than in the previous week. The message is translated freely from the Dutch version

“We are concerned about your eating problems. You indicate that last week you have been worrying about food and your body, you have not eaten sufficiently every day and have had more days on which you dealt with binges and compensatory behaviors. That’s no small thing you’re dealing with :(.	
The urge to eat can emerge from stress, tension and/or emotions that suddenly occur. Is that something you recognize? Do one or more of these factors also precede a binge for you? It is possible to directly respond to these tensions or emotions by giving in to your binge. However, in fact you are not really heeding them, but you are muffling or dampening them and putting them aside. This mostly has a reversed effect, since not only do these tensions and emotions return at a later time, you generally feel worse after a binge as well.	
Next time you feel an urge to binge or compensate your food, try to delay it. You will notice that after a while the binge or compensating behavior seems less necessary, or even not necessary at all! For this week, try to delay the urge for about 10 minutes. Also think about activities you can undertake during those 10 minutes to make delaying your binge or compensating behavior more bearable. Call a friend, put on your favorite music, go on a stroll through town or find another activity. Did you achieve the 10-minute delay? Excellent! Challenge yourself to extent the time you set for yourself every now and then.	
Will you rise to the challenge? We are very curious to see what will happen when you learn to delay your harmful eating behaviors and whether this will help you. Good luck!”	

#### Weekly expert-patient support

All recruited expert patients (*N* = 5) have participated in an intensive day of training. The first part of the training comprised how to use the experience of having had and overcoming an ED to help others struggling with ED-related problems. The second part comprised an elaborate explanation of the research and the Featback program. Subsequently, a training specifically focused on the delivery of online support via chat and e-mail was delivered. An intervention protocol was handed out and explained to the expert-patient support team. The protocol includes guidelines about how to provide support so that all expert patients will work from a similar perspective and with similar methods. The five-phase model on which the intervention is based contains (1) warm welcome, (2) clarify the question, (3) determine the goal of the conversation, (4) elaborate on the goal of the conversation, and (5) close the circle. The phases of e-mail support are (1) extract the question, (2) formulate an answer, and (3) check and send the message. More detailed information on the models for e-mail and chat support can be found in the handbook written by Schalken et al. [56]. The expert-patient supporters have practiced with offering chat and e-mail support during and after the training, and feedback on their practice sessions was provided by an expert patient and experienced psychologist. Participating expert patients will receive monthly supervision during the study by an experienced expert patient and clinical psychologist to ensure high-quality and ethically correct support. They have a set amount of hours per week that they can flexibly distribute, and they receive monthly payment for worked hours on the project.

Participants can schedule a weekly appointment with an expert patient. For each session, participants can choose to receive support via chat or e-mail. Chat sessions have a duration of 20 min, and for e-mail support participants are required to send an e-mail before the scheduled appointment to which an expert patient will reply at the time of the appointment.

#### Waiting list control

Participants will be placed on a waiting list for 14 months (matching the participation duration of participants in the other conditions; 8-week intervention period plus 1 year follow-up), after which they will be offered 8 weeks of Featback with support from an expert patient. Participants in this condition will be asked to complete the same assessements as participants in the other conditions (i.e., T0-T5). Note that participants in all conditions are allowed to seek and receive treatment and take medication.

#### Intervention check

To assess the difference between support by expert patients and psychologists, a formal integrity check will be conducted. After data collection, 15 randomly selected chat and 15 e-mail sessions of expert patients will be compared to 15 randomly selected chat and 15 e-mail sessions of psychologists respectively (taken from the previous RCT of this research group; 20). Subsequently, three independent master level psychology students, blind to the source of the e-mail or chat session, will rate the 60 sessions with the integrity list. Expert patients and psychologists collaborated to create the integrity checklist, which involves (1) the structure of the session, (2) the content/interventions used during the session, and (3) the way in which these interventions were conveyed (see Additional file 2). We expect that the structure of an e-mail or chat is similar between expert patients and psychologists, since the same structuring methodology is used. However, psychologists are expected to use a broader pallet of interventions (i.e., more distinct interventions) during the e-mail and chat support. Lastly, the most noticeable difference is expected in the way in which interventions are conveyed. More specifically, it is hypothesized that expert patients will explicitly mention their own experiences during every e-mail or chat session to try to change attitudes or behaviors of participants, whereas psychologists will never do this. Additionally, expert patients are expected to use fewer medical terms or abbreviations in their e-mail or chat sessions than psychologists.

### Measures

Table 2 presents an overview of the assessment instruments used for each measurement time. Estimated times (minutes) to complete each questionnaire are presented in parentheses. Details of the instruments are described in the following sections.

**Table 2:**
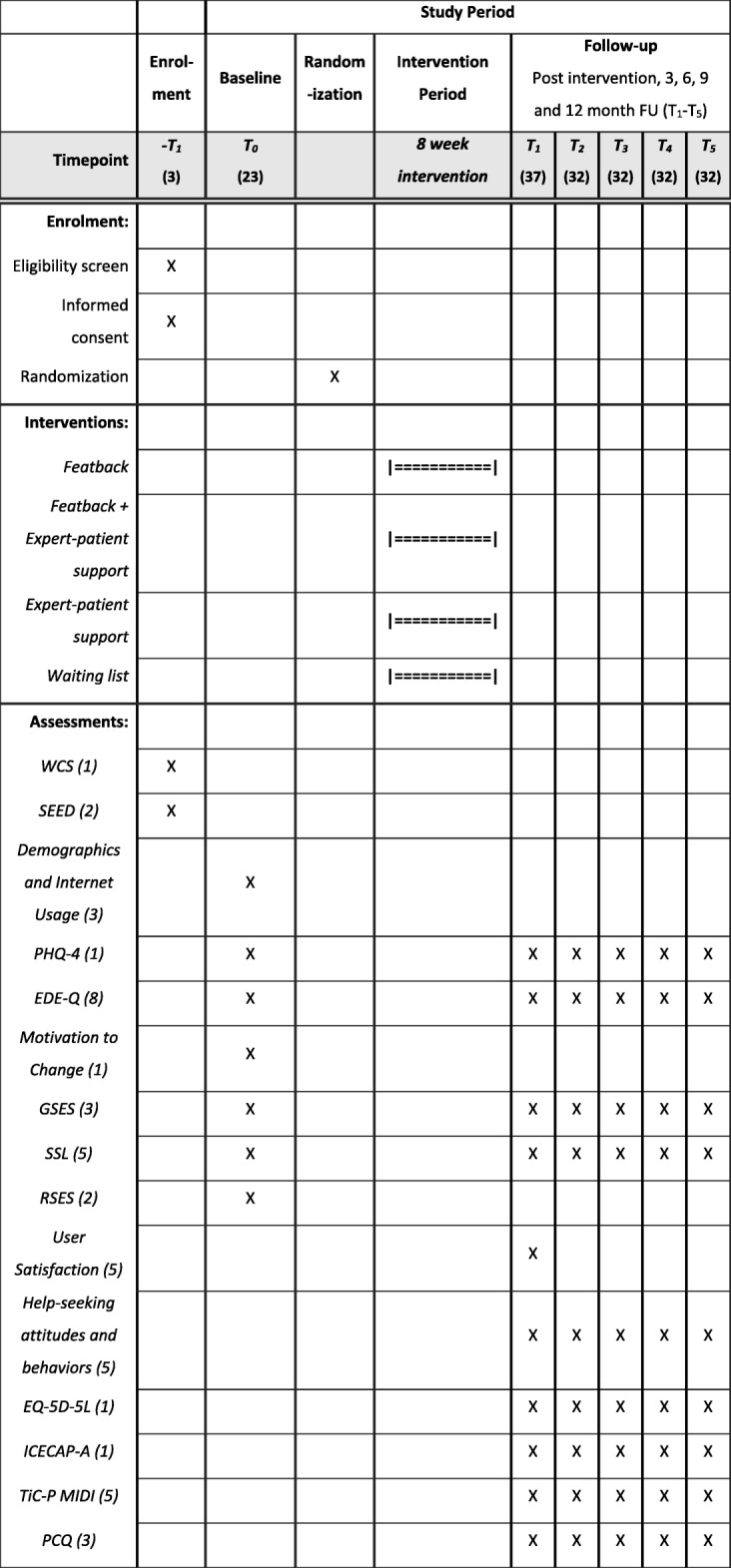
Overview of assessment occasions and their content

*Note*: The Inclusion of Other in the Self scale (IOS scale; 0.5 min to complete) will be sent at week 3 of the intervention for all participants in a condition with expert-patient support. Attrition follow-up questions will be sent only to participants who do not respond to the assessments

*FU* follow-up, *WCS* Weight Concerns Scale, *SEED* Short Evaluation of Eating Disorders, *PHQ-4* Patient Health Questionnaire, *EDE-Q* Eating Disorder Examination Questionnaire, *GSES* General Self-Efficacy Scale, *SSL* Social Support List, *RSES* Rosenberg Self-Esteem Scale, *EQ-5D-5 L* EuroQol five dimensions, five levels generic health index, *ICECAP-A* ICEpop CAPability measure for Adults, *TiC-P MIDI* Trimbos/iMTA questionnaire for Costs associated with Psychiatric Illness Midi version, *PCQ* Productivity Costs Questionnaire

#### Screening measures

##### Weight Concerns Scale

The Weight Concerns Scale (WCS [54]) is a five-item questionnaire used to evaluate the eligibility of participants. The five items are derived from a principal component analysis of a list of questions used to measure ED symptoms [54] and assess the extent to which participants struggle with their weight, eating pattern, shape, and perceived corpulence. Test-retest reliability and predictive validity have been investigated and demonstrated for the WCS [57]. Furthermore, the WCS has been found to be able to predict students at risk for developing an ED [58].

##### Short Evaluation of Eating Disorders

The Short Evaluation of Eating Disorders (SEED [55]) contains six self-report questions designed to quickly assess the key ED symptoms. Participants are asked to evaluate their own body on several dimensions (e.g., thinness, attractiveness, and muscularity) and to report the frequency of several ED-related behaviors, such as self-induced vomiting, use of laxatives, and binge eating, over the last 4 weeks. Items are presented on a 5-point Likert scale. Summing the items of the two separate diagnoses leads to a severity index (range 0–3), with higher scores indicating higher severity. The SEED has been found to have good construct and criterion validity and was demonstrated to be sensitive to symptom change [55].

#### Primary outcome measures

The primary outcome measures involve the Eating Disorder Examination Questionnaire (EDE-Q [59]), which will be used to assess ED symptomatology. The EDE-Q has 28 items and assesses both the frequency of core ED behaviors (6 items) and ED-related attitudes (22 items) over the past 28 days. Items assessing the ED-related attitudes are presented on a 7-point Likert scale (range 0 ”not at all” to 6 ”every day/markedly”) and include questions regarding weight, shape, and eating concerns and restraint. A global ED psychopathology score will be calculated by summing and averaging the 22 items. Higher scores indicate higher ED psychopathology. Internal consistency, test-retest reliability, and discriminative validity of the EDE-Q have been found to be acceptable to high [60].

#### Secondary outcome measures

##### General Self-Efficacy Scale

The General Self-Efficacy Scale (GSES [61]) is a 10-item psychometric scale designed to measure a general sense of perceived self-efficacy with the aim to predict coping with daily hassles and adaptation after experiencing various stressful life events. The questionnaire has been used in many studies with numerous participants [62]. Responses are recorded on a 4-point scale. The individual items are summed to produce the final composite score with a range of 10 to 40. In samples from 25 nations (including the Netherlands), Cronbach’s *α* ranged from 0.75 to 0.91, with most values over 0.80. Psychometric properties of the GSES are adequate [63]. The scale is one dimensional and designed for the general adult population, including adolescents.

##### Social Support List

Perceived social support is measured with the short version of the Social Support List Interaction (SSL-12-I [64]). This self-report questionnaire measures the extent to which a participant experiences social support. The SSL-12-I contains 12 items in three scales, namely (1) everyday social support, (2) support in problem situations, and (3) esteem support (i.e., support resulting in self-esteem). Items (starting with ”Does it ever happen that people…” and ending with statements like ”…comfort you?” or ”…give you good advice?”) are presented on a 4-point scale ranging from ”hardly or never” to ”very often”. Scores range from 12 to 48, and higher scores are indicative of more experienced social support. Psychometric properties are demonstrated to be good [64, 65].

##### Patient Health Questionnaire

The Patient Health Questionnaire (PHQ-4 [66]) measures symptoms of depression and anxiety. It consists of two primary anxiety items and two primary depression items. The anxiety and depression subscales have been found to reflect two separate dimensions [66]. The four items are presented on a 4-point Likert scale ranging from 0 ”not at all” to 3 ”nearly every day”. By summing all items, a composite score (range 0–12) can be calculated. Higher scores indicate higher pathology. The PHQ-4 has been demonstrated to possess factorial and construct validity [66].

##### Motivation to change

Three items will be used to assess participants’ motivation to change [67, 68]. The first item assesses the perceived importance to change of participants (*On a scale of 1 to 10, how important is it for you to change?*)*.* The second item assesses the ability or confidence to change (*On a scale of 1 to 10, how confident are you that you could make a change if you wanted to?*)*.* The third item assesses one’s readiness to change (*On a scale of 1 to 10, how ready, or how prepared are you to change? Are you not prepared to change, already changing, or somewhere in the middle?*)*.*

##### User satisfaction

To assess the user satisfaction of Featback, a questionnaire was developed. Among other questions, participants are asked how they rate the quality of support they have received from Featback, whether Featback helped them to more effectively cope with their eating-related problems, and the extent to which they were satisfied with Featback in general. Additionally, participants are requested to rate the various components of Featback and address positive points as well as points for improvement (e.g., they are asked what they liked or disliked most and how the intervention can be developed further).

##### Help-seeking attitudes and behavior questionnaire

A custom-made questionnaire was developed to assess help-seeking attitudes and behaviors. Participants are presented with three 7-point Likert scale questions (ranging from 0 ”not at all applicable” to 6 ”fully applicable”) concerning the extent to which they believe professional help is useful, they need professional help themselves, and they know where to find help. Furthermore, intention to seek professional help and actual help-seeking behavior are assessed, and the extent to which Featback contributed to these processes is inquired. Depending on whether the participant has sought help or not, the number of questions in this section ranges from three to five; they are either open or yes-no questions. Finally, participants are inquired as to the frequency of visiting websites other than Featback in relation to their (eating) problems, visiting and/or using a forum, or making use of online support service in relation to their (eating) problems.

##### Intervention usage

Intervention usage will be operationalized by the amount of weekly monitoring assessments a participant has completed during the intervention period (range 0–8). Additionally, to be able to further investigate the relation between intervention usage and the effectiveness of Featback, the number of received support sessions of participants (one for each received e-mail or chat session) will be recorded.

#### Cost-effectiveness measures

##### General quality of life

The EuroQol five dimensions, five levels generic health index (EQ-5D-5 L [69]) is a standardized self-report questionnaire consisting of five dimensions (mobility, self-care, usual activities, pain/discomfort, and anxiety/depression). Participants rate each dimension on five levels (ranging from ”no problems” to ”extreme problems”). Consequently, 243 distinct health states are defined, each with a unique utility score, ranging from 1 (perfect health) to 0 (death). The health descriptions will be linked to empirical valuations of the Dutch general public, allowing utilities to be computed [70].

While the EQ-5D-5 L is the gold standard for computing utilities and economic evaluations, it is limited in the sense that it mainly addresses physical aspects of the health experience and might not be appropriate for assessing mental health problems. For example, psychiatric patients may not endure many physical problems while still experiencing considerable distress. Approaching outcomes for individuals by focusing on people’s capabilities, instead of physical aspects of health, might therefore be more suitable for individuals with psychiatric conditions [71]. The ICEpop CAPability measure for Adults (ICECAP-A [72]) shows promise in going beyond the general health status and capturing broader outcomes for individuals. The questionnaire aims to measure five capabilities on a 4-point scale, namely stability (the extent to which someone feels consistency and safety in life), attachment (the extent to which someone feels love, friendship, and support in life), autonomy (the extent to which someone can be independent in life), achievement (the extent to which someone can make progress in life), and enjoyment (the extent to which someone can enjoy life). The five items attempt to capture an individual’s capability to live a life that he/she values. The ICECAP-A has been found to be a valid measure of one’s capabilities [73] and appears to be suitable for economic evaluation of outcomes of adults with mental health problems, such as depression [71].

##### Direct medical costs

The Trimbos/iMTA questionnaire for Costs associated with Psychiatric Illness (TiC-P, Midi version [74]) will be used to calculate the total direct medical costs. The TiC-P assesses medical treatment utilization (e.g., number of contacts with the general practitioner, medical specialists, and paramedics) and medication use during the last 3 months. Additionally, the Midi version of the questionnaire is significantly shorter, reducing the burden for participants, while retaining 90% of the total cost estimated by the full version [74]. The direct medical costs will be calculated using the Dutch guidelines for cost calculations in healthcare [75]. Reference unit prices of the corresponding health care services will be applied [75, 76].

#### Demographics, closeness, self-esteem, and attrition

##### Demographic

A self-designed questionnaire will be used to gather demographic information. More specifically, gender, age, educational level, country of origin, and work situation will be assessed. Three additional questions are included, to inform about Internet access, the severity of eating problems, and whether participants have previously been or are currently in treatment for an ED.

##### Inclusion of Other in the Self scale

The Inclusion of Other in the Self scale (IOS scale [77]) will be sent to participants allocated to one of the conditions with support by an expert patient at week 3 of the intervention to assess perceived similarity and feelings of closeness early in the working relationship between expert patient and participant. The IOS scale is a one-item instrument in which seven images with two circles representing the self and the other that overlap increasingly, from zero overlap to almost complete overlap, are presented to participants. The participants can then rate their relationship with another person by choosing the best fitting image. More overlap between the circles indicates a closer bond. This scale is particularly suited for the current study, since it can be used for any type of relationship, and not only romantic partners, friends, and acquaintances [78]. Furthermore, Gächter, Starmer, and Tufano [79] conducted three studies to examine the validity and reliability of the IOS scale. A very strong convergent validity with a closeness index derived from six other relationship inventories (Spearman correlation of 0.85) was found. The authors conclude that the one-item IOS scale is easy to use, highly reliable, and a very powerful measure of closeness of relationships.

##### Rosenberg Self-Esteem Scale

The Rosenberg Self-Esteem Scale (RSES [80]) is the most used measure of global self-esteem. It consists of 10 items measuring the affective evaluation of the self. The items are presented on a 4-point scale ranging from ”totally agree” to ”totally disagree”. The questionnaire has been found to have satisfactory psychometric properties [81]. The Dutch translation of the RSES originates from Franck, De Raedt, Barbez, and Rosseel [82], who created the translation using forward and back translation methods. Two studies indicate high internal consistency and good convergent and divergent validity of the Dutch version of the RSES [82, 83].

##### Attrition follow-up

Attrition can refer to participants no longer using the intervention (non-usage attrition) or to participants completely dropping out of the study (dropout attrition). Two questions were designed to investigate why people dropped out of the study or no longer used the intervention to which they were allocated.

If a participant fails to complete a monitoring assessment, an e-mail with a reminder will be sent. A week later, participants receive another reminder. This reminder includes a question asking whether one wishes to continue Featback or not, and, if not, participants are asked to answer one more question by writing down their reason(s) to quit the intervention (i.e., attrition follow-up question).

If a participant fails to complete a T1, T2, T3, T4, or T5 assessment within 1 week, an e-mail with a reminder will be sent. A week later, a second reminder will be sent. This reminder includes a question asking whether one wishes to further participate in our study, and, if not, participants are asked to answer one more question by writing down their reason(s) to quit the study.

### Participant procedures

Figure 1 depicts an overview of the procedures that participants will undergo throughout the study. After recruitment, interested individuals can send an e-mail to the main researcher. Consequently, they will be sent a reply in which they are thanked for their interest and invited to complete a screening questionnaire. Participants who complete the screening questionnaire receive an e-mail including feedback on their results and a notification about whether or not they are considered eligible for the study.

Participants who are eligible for the study will be sent an e-mail which contains an explanation of the study and corresponding procedures. Participants then have to give their informed consent to continue with the study. Agreeing to the terms of participation will be possible through clicking several checkboxes. Because informed consent is given online, the system will generate a pop-up at the end of the form with a question asking participants are sure they give consent to participate in the study. Here, participants will also be notified that they can leave the study at any time for any reason if they wish to do so, without any consequences. Subsequently, participants are presented with a link to the first assessment (T0). After giving informed consent and filling in the baseline measurement, participants will be randomized to one of the four conditions and will receive an e-mail about the condition to which they are allocated and the corresponding procedures.

At this point, the 8-week period of intervention (or waiting) starts. At post-intervention (T1, week 8) and for all follow-up measurements (T2, 3 months; T3, 6 months; T4, 9 months; T5, 12 months) participants will be asked to complete the corresponding questionnaires. Finally, participants will receive an e-mail in which they are thanked for participating in the study. Participants who take part in the study receive 10 euros as compensation in the form of a gift voucher after the last measurement. Additionally, participating in the study will be made more personal and rewarding in the form of an e-mail sent to participants after every T1–T5 assessment, in which gratitude for participation is expressed. Hopefully, these incentives increase intervention usage and reduce attrition in the study.

### Ethical considerations

Participants will be asked to complete questionnaires at baseline, a weekly monitoring questionnaire during the intervention, and a three-monthly questionnaire during the follow-up phase. This has been found to be an acceptable burden by a client panel. Earlier research with a comparable design showed no adverse effects of Featback [20], and participants in all conditions, including the waiting list, are allowed to seek treatment outside the study.

Individuals who enter the study and report severe ED symptoms during the screening or who develop severe ED symptoms during the intervention will be sent an e-mail stating that their scores indicate serious ED problems and that professional help is warranted. More specifically, if a participant indicates a BMI of 15 or lower or reports compensatory behavior or bingeing at least every day during a week in the screening or Featback monitoring assessment, an alarm signal will be sent to the main researcher. If the participant is allocated to the Featback only or waiting list control condition, the participant will be sent an e-mail in which the researchers’ concerns about the severe ED symptoms are expressed and recommendations for professional help are included. If the participant is allocated to one of the expert-patient support conditions and the participant has not scheduled an appointment for this week, an e-mail will be sent to encourage the participant to schedule an appointment. During the support session, the expert patient will discuss the alarm signal and severe ED symptoms and stimulate the participant to seek professional help. Similarly, participants in the support only condition who indicate (increasingly) severe problems in their chats or mails will be encouraged to seek professional help in subsequent sessions. If participants with severe ED symptoms do not make an appointment for a support session, the e-mail as described for participants in the Featback only or waiting list control condition will be sent.

Nevertheless, individuals with severe ED symptoms will not be excluded from the study, as there is no reason to withhold Featback or expert-patient support. It could well be that these individuals are reluctant to seek (face-to-face) treatment or that they are not fully aware of the severity of their symptoms. Accordingly, Featback may serve as an important first step to regular healthcare, because it could help individuals with the process of recognition and acknowledgement of the severity of their ED symptoms and the need to seek professional help (see also [18]). Moreover, Featback and/or the individualized support from expert patients may serve as an important and unique source of support that could help individuals deal with their (eating) problems more effectively.

Expert patients are instructed to refer participants who report suicidal ideation to the website of ”Stichting 113” [84]. The goal of this organization is to prevent suicide. They have psychologists, psychiatrists, and trained volunteers in employment who are accessible 24 h a day via telephone and chat.

### Sample size calculation

An a priori statistical power analysis was conducted in G*Power version 3.1 to determine the optimal sample size having 80% power to detect a small effect size (*f* = 0.15, which corresponds to *d* = 0.30) between the active intervention conditions on the one hand and the control condition on the other. Consequently, the sample size calculation was based on a between-factors repeated measures analysis of variance (ANOVA) with two groups and two measurements (i.e., baseline and post-intervention; T0–T1). A significance level of α = 0.05 was maintained. The effect size was based on data from a previous RCT [20]. Calculations indicated a total of 264 participants will be needed, meaning 88 participants per condition. We assume a Pearson correlation of 0.5 between the outcome variable on baseline and post-intervention (i.e., T0–T1), which explains 25% of the variance of the outcome variable. Therefore, the sample size per group can be reduced by 25%. However, adjusting for an anticipated dropout rate between T0 and T1 of 25% (based on previous data), we will still need 88 participants per condition (*N* = 352). The high dropout rate introduces a risk of bias through selective dropout. However, participants in the previous Featback trial [20] who dropped out during the intervention did not differ from those who did not drop out with regard to ED psychopathology, ED quality of life, comorbid anxiety or depression, age, weight, hours spent online, allocated condition, and duration of their eating problems. Hence, bias because of selective dropout in the current sample is, at least based on these variables, improbable.

### Statistical analyses

All statistical analyses will be conducted in SPSS version 25 and the R statistical programming environment [85]. A two-tailed significance level of *α* = .05 will be maintained throughout the analyses unless indicated otherwise. All analyses will be conducted according to the intention-to-treat (ITT) approach. This means that all participants who underwent randomization, even those who withdrew from the study or deviated from the protocol, are included in the analyses. For the effectiveness analyses, both ITT and completers analyses will be conducted. A participant is considered a completer when he/she has completed at least five monitoring assessments (i.e., Featback only condition), five support sessions (i.e., expert-patient support only condition), or both (i.e., Featback plus expert-patient support condition).

Missing data will be handled using multiple imputation [86]. Multiple imputations using predictive mean matching will be conducted in the statistical programming environment R [85]. Interactions will be taken into account in the imputation procedure [87]. Multiple imputation methods have several advantages over complete-case analyses or single imputation techniques and are therefore highly recommended [86, 88].

Homogeneity of groups (i.e., between-group differences) will be assessed at baseline (T0) using chi-square tests for categorical variables and ANOVAs for continuous variables. Additionally, homogeneity of variances will be assessed using Levene’s statistic, and non-parametric testing will be used when appropriate.

#### Intervention effectiveness analyses

To investigate the effectiveness of Featback with and without weekly expert-patient support, within-group and between-group effect sizes (Cohen’s *d*) will be calculated (i.e., the effects of time and intervention) using the pooled standard deviation of each group. We are mainly interested in the effect from baseline to post-intervention (T0–T1), but to see if effects are maintained over the short and long term, analyses will be repeated for T1–T3 and T1–T5 respectively.

1. To answer the first and main hypothesis that the active interventions (i.e., Featback, expert-patient support, and Featback plus expert-patient support) are more effective than a waiting list in reducing ED symptomatology (i.e., primary outcome EDE-Q global score), the three active intervention conditions will be compared to the waiting list condition. Repeated measures ANOVA will be used for T0–T1 to test this hypothesis. To see if the effects are maintained, these analyses are repeated for T1–T3 and T1–T5.
2. The second hypothesis was that the combination of Featback and expert-patient support would be more effective than Featback or expert-patient support alone. A repeated measures ANOVA with post hoc tests will be conducted to compare T0–T1 differences in ED symptomatology between the four conditions. The post hoc analyses apply the Bonferroni correction for multiple testing. To see if these effects are maintained, these analyses are repeated for T1–T3 and T1–T5.

These confirmatory analyses will be repeated controlling for significant baseline variables (i.e., age, duration of ED psychopathology, number of psychological healthcare appointments). Additionally, the main analyses will be repeated for completers only.

#### Moderator and mediator analyses

Potential moderators of treatment effects will be investigated using model-based recursive partitioning methods [89]. Model-based recursive partitioning can be used to detect what are called treatment-subgroup interactions. Treatment-subgroup interactions occur when subgroups of patients show differences in the effectiveness (i.e., a better or worse outcome) of one or more interventions. Model-based recursive partitioning can be used to identify these subgroups and their characteristics [90, 91] and ultimately help to tailor treatment to individual patients. These analyses will be explorative in nature and will apply the conservative Bonferroni correction for multiple testing.

(H3) The third hypothesis concerned the relationship between the closeness or perceived similarity of participants with the expert patient they are assigned to, and ED symptomatology, self-efficacy, and experienced social support respectively. The effects of perceived similarity on the three dependent variables will be investigated for the short term (i.e., gains until post-intervention, T1) and the long term (i.e., gains until the last follow-up, T5), resulting in six linear regression analyses that need to be conducted. We correct for multiple testing for these analyses using Holm’s method.
(H4) To investigate the fourth hypothesis that changes in self-efficacy during the 8-week intervention period mediate subsequent changes in ED-related symptoms and experienced social support (and not vice versa), a cross-lagged panel design will be used. For self-efficacy and the two outcome variables, change scores will be calculated for pre- (T0) to post-intervention (T1) and for post-intervention (T1) to long-term follow-up (T5; 12 months after T1). Next, hierarchical regressions will be performed, with post-intervention to long-term follow-up change of ED-related symptoms and experienced social support as dependent variables, and pre- to post-intervention change of self-efficacy as the independent variable. Additionally, the inverse relationships will be investigated. In other words, it will be examined whether changes in experienced social support or ED-related symptoms from pre- to post-intervention can predict post-intervention to long-term follow-up changes of self-efficacy. If the initial relationship is significant and the inverse relationship is not, a mediation effect of self-efficacy on ED-related symptoms and/or experienced social support is indicated. Corrections for autocorrelation and synchronous change will be applied to the mediation analysis.

#### Satisfaction and intervention usage analyses

(H5) To examine the fifth hypothesis that Featback with weekly expert-patient support results in an increased satisfaction and intervention usage, an ANOVA will be conducted to compare mean scores of satisfaction and intervention usage at T1 (i.e., directly after the intervention) between the three active intervention conditions. We correct for multiple testing for these analyses using Holm’s method.

#### Cost-effectiveness analyses

The effects and costs of Featback and/or online support from an expert patient will be compared to those of usual care (i.e., participants in the waiting list condition) from a societal perspective, including healthcare costs and non-healthcare costs. No discounting will be applied due to the time horizon of the economic evaluation of 1 year.

The effects of an intervention will be assessed with the EQ-5D-5 L at baseline and subsequent follow-up measurements. The EQ-5D-5 L results will be translated into utilities using the EQ-5D-5 L with Dutch rates [92]. The quality-adjusted life year (QALY) outcome per patient will be obtained by using the area-under-the-curve method for the utility scores obtained for each patient.

Outcomes of patients in terms of capabilities instead of QALYs will also be calculated using the ICECAP-A by means of the UK general population tariff (no Dutch tariff is available) [93], since results from this instrument might reflect outcomes of psychiatric patients better than the EQ-5D-5 L results [71].

The costs will be divided into healthcare costs and non-healthcare costs. Healthcare costs are calculated by summing the costs of Featback and/or online support from an expert patient, and other healthcare use during the first year of follow-up. Intervention costs of Featback include the maintenance of the program and website and payment to a psychologist following the procedure when a user develops severe ED pathology while using Featback. Intervention costs of expert-patient support are estimated by multiplying the time spent on sessions by their hourly pay rate. Costs for supervision of expert patients are also included, by multiplying the time spent at supervision by their hourly pay. Healthcare use is assessed with the TiC-P Midi [74], which records the number of contacts with care providers and use of medication during the last months (i.e., the period between every follow-up measurement). The costs will be calculated by multiplying the number of contacts with a specific healthcare provider by the reference unit price of the corresponding healthcare service [75, 76]. Additionally, non-healthcare costs, including costs related to productivity losses at work through being absent or being less productive and having difficulties in performing unpaid work such as domestic tasks, are estimated. Costs related to absenteeism will be calculated according to the friction cost method, which means that the absent hours are multiplied by the average gross hourly wage per paid working individual in the Netherlands with a maximum of 12 weeks, the friction period in the Netherlands [75, 76]. The friction period is the timespan organizations need to restore the initial production level [94]. Costs related to reduced efficiency at work are calculated based on the amount of hours of work participants estimate they need in order to catch up for all the work they were unable to perform because of health problems. These hours are again multiplied by the average gross hourly wage per paid working individual in the Netherlands, based on age and gender. Costs related to difficulties in performing unpaid work are calculated by multiplying the amount of hours that others would need to take over the unpaid work of the participant by the average gross hourly wage of a domestic worker. In summary, total costs of an intervention can be estimated by summing the healthcare costs (including the intervention costs) and non-healthcare costs consisting of productivity costs (including absenteeism, reduced efficiency at work, and difficulties performing unpaid work).

Differences in mean costs and effects per patient between interventions will be compared using a two-sided *t* test. The uncertainty regarding mean costs and effects per participant will be estimated using bootstrapping in Microsoft Excel, simulating 1000 bootstrap samples. Specifically, 1000 samples will be drawn from the original sample to estimate the sampling distribution and its 95% confidence interval. The results of the bootstrapping will be represented in cost-utility acceptability curves. These curves illustrate the probability that an intervention (i.e., Featback) is cost-effective in comparison with the alternative (i.e., care as usual) for a range of ceiling ratios, which are the maximum amount of costs a society is willing to pay for one unit change in outcome (i.e., QALY).

### Data management

Data of participants will be handled and saved strictly confidentially according to the enforced laws and regulations, including the EU General Data Protection Regulation (GDPR) and the Declaration of Helsinki – 64th WMA General Assembly, Fortaleza, Brazil, October 2013. Data obtained from participants will be, among others, e-mail address, age, level of education, and data about the health of participants. Participants’ e-mail addresses and other data that can be directly traced to them will be coded with a number so that their privacy is protected. Non-coded data will be saved separately, and only the main researchers, the accredited METC, and Inspectie Gezondheidszorg en Jeugd (IGJ) will have access to this data file. Data will be kept for a minimum of 10 years, according to guidelines from the Association of Universities in the Netherlands (VNSU). Participants can withdraw from the study at any moment without consequences. Data gathered from participants up until their withdrawal will still be used for analyses. No official data monitoring committee will be formed, since no difficulties in data management are anticipated, but use of a data log, making back-ups of the anonymized data file, and regularly checking data completeness are several methods that will be employed to promote data quality.

## Discussion

The aims of the current study are threefold. The first aim is to investigate the (cost-)effectiveness of the Internet-based self-help program Featback with and without expert-patient support. The second aim is to explore predictors, moderators, and mediators of intervention response, to better understand how and for whom Featback works. Thirdly, practical experiences with Featback, such as the intervention usage and user satisfaction, will be examined.

The current study design has several strengths. Firstly, it is highly similar to a previous RCT from the same research group. Therefore, findings regarding the effectiveness of Featback can be replicated, and limitations of the previous study can be overcome. Secondly, cost-effectiveness analyses of Internet-based interventions are rare but very useful in judging which interventions should be applied over others. Indeed, with ever-declining finances for health provisions and an increase in desire to offer effective and inexpensive treatment by health insurances, cost-effective analyses are indispensable. By conducting such an analysis, the current study aims to contribute to economically sensible choices regarding treatment for individuals with eating-related problems that are found to be effective as well. Thirdly, the ITT approach used for data analyses and the multiple imputations used for handling missing data are solid and recommended methods. Lastly, the relatively long follow-up period of 1 year helps to more fairly examine the effectiveness of the different interventions in the long term.

Additionally, some limitations of the current study design should be noted. Firstly, participants are allowed to engage in treatment outside of the research for ethical reasons. Although we will control for healthcare appointments during the analyses, methodologically it would be preferable to have all the participants only receive the experimental intervention. Lastly, only online measures will be completed by participants. This limits the diagnostic accuracy, introduces recall bias, and might reduce intervention usage as well. Indeed, we expect a fairly high dropout rate, and missing data might not be at random, which will need to be taken into account when analyzing the data. Nevertheless, the current approach is needed to maintain the low threshold and anonymity of the intervention, making it possible to generalize beyond this study to the real effects of Featback with and without expert-patient support.

The (cost-)effectiveness of Internet-based interventions in combination with expert-patient support for the (early) interventions of individuals with an ED or related symptoms has not been investigated before. Results on this subject will contribute to the delivery of e-mental health and expert-patient support and help to guide individuals with eating problems to the care they need.

### Trial registration

This is protocol version number 2, dated 02-28-2019. Recruitment started 8 October 2018. The approximate date of recruitment completion is 31 December 2020. The trial is registered in the Netherlands Trials Register under number NTR7065.

## Additional files

Additional file 1: SPIRITchecklist. (DOC 120 kb)

Additional file 2: Integrity checklist. (DOCX 23 kb)

## Data Availability

The datasets generated and analyzed during the current study are available from the corresponding author on reasonable request, and in the DANS repository after study completion.

## Abbreviations

ED: Eating disorder
EDE-Q: Eating Disorder Examination Questionnaire
EQ-5D-5 L: EuroQol five dimensions, five levels generic health index
FU: Follow-up
GSES: General Self-Efficacy Scale
ICECAP-A: ICEpop CAPability measure for Adults
IOS scale: Inclusion of Other in the Self scale
PCQ: Productivity Costs Questionnaire
PHQ-4: Patient Health Questionnaire
QALY: Quality-adjusted life year
RCT: Randomized controlled trial
RSES: Rosenberg Self-Esteem Scale
SEED: Short Evaluation of Eating Disorders
SSL: Social Support List
TiC-P: Trimbos/iMTA questionnaire for Costs associated with Psychiatric Illness
WCS: Weight Concerns Scale
