# Supplementary material for: A randomized controlled trial of an Internet-based intervention for eating disorders and the added value of expert-patient support: study protocol
Source: Trials. 2019 Aug 16;20:509. doi: 10.1186/s13063-019-3574-2 (PMC6697984; doi:10.1186/s13063-019-3574-2)
Supplement: Supplementary file 2 — Integrity checklist. (DOCX 23 kb) [file 13063_2019_3574_MOESM2_ESM.docx]

**Integrity Checklist Psychologist/Expert Patient Sessions**

The current session is an EMAIL / CHAT

1. **Structure**

The following elements should ALL be present in an email or a chat session:

| **E-Mail** | **Chat** |
| --- | --- |
| 1.Extraction of the question  2.Formulation of an answer  3.Ending | 1.Warm welcome  2.Establishing the topic  3.Establishing what will be discussed in the current chat  4.Discussing the topic / conveying support or advice  5.Ending |
| All present? YES / NO | All present? YES / NO |

1. **Content/Interventions**

The aim of the intervention is to make people aware of their eating problems and to provide ways/suggestions to enlarge this insight, counteract eating related problems and/or to stimulate seeking help.

NOTE. If a method/delivery/intervention falls under more categories it only counts as one (no double counts).

| The session took at least 20 minutes | YES / NO |
| --- | --- |
| The situation was assessed / summarized for participants | YES / NO |
| The topic of conversation was established | YES / NO |
| Count the interventions present in the form of:   1. Giving support / empathy 2. Reflecting feelings 3. Motivating 4. Expressing concern 5. Asking for more clarity | AMOUNT  1  2  3  4  5 |
| Count the interventions present in the form of:   1. Providing Psychoeducation 2. Providing advice 3. Concretizing aims or goals 4. Stimulating thinking / reflection 5. Confronting 6. Challenging cognitions / beliefs 7. Suggesting to seek help / treatment 8. Explain procedures 9. Other… | AMOUNT  1  2  3  4  5  6  7  8  9 |
| At the end of the session   1. the participant knows what to do in the short term (coming week) 2. concrete advice or directions are provided by the supporter 3. suggestion(s) about dealing with obstacles or difficulties is/are provided | YES / NO  YES / NO  YES / NO |
| NUMBER OF ‘YES’ (range 0-6)  NUMBER OF INTERVENTIONS (sum of interventions) |  |

1. **Method of Delivery**

Contents the way in which interventions are delivered to the participant.

NOTE. If a method/delivery/intervention falls under more categories it only counts as one (no double counts).

| A. Interventions were present in the form of:   1. Sharing (common) knowledge or scientific findings to introduce or complement advice or psychoeducation 2. Sharing (common) knowledge or scientific findings to show support or to reduce stigma/feelings of shame (“many people with eating problems…”) 3. Presenting solutions to problems by mentioning (directly) that is has been found to work in research or by other people (“… works for many people with eating problems”) 4. Sharing one’s own experience in a way that a participant feels recognized / to break stigmatization / to give hope to participants 5. Sharing one’s own experience to offer advice 6. Sharing one’s own experience to stimulate seeking help or treatment 7. Sharing one’s own experience to offer psychoeducation | YES / NO  YES / NO  YES / NO  YES / NO  YES / NO  YES / NO  YES / NO |
| --- | --- |
| B1 At the end of the session it is evident that the supporter is a person with knowledge about the problems the participant is currently struggling with and maintains a psychologist approach  B2 At the end of the session it is evident that the supporter has had experience with (a form of) the problems the participant is currently struggling with | YES / NO  YES / NO |
| C1 The supporter never talks about his/her own life (e.g. a situation, feeling/emotion, thought(process), difficulty, success) during the session  C2 The supporter has revealed something (e.g. a situation, feeling/emotion, thought(process), difficulty, success) about his/her life as a tool to offer support to the participant during the session (can overlap with the first check of this table) | YES / NO  YES / NO |
| D The supporter never uses medical terminology or medical abbreviations (or if it used: explains what the term/abbreviation means or verifies that the participant knows what the term/abbreviation means) | YES / NO |

| E1 I (the rater) believe the supporter is a psychologist  E2 I (the rater) believe the supporter is an expert patient | YES / NO  YES / NO |
| --- | --- |

| ***Integrity – Final Score*** | | | |
| --- | --- | --- | --- |
| **A** | **Structure** |  | YES / NO |
| **B** | **Content** | Number of interventions used | … |
|  |  | Number of YES (range 0-6) | … |
| **C** | **Method** | Number of YES in A1-3, B1, C1, E1 (range 0-6) | … |
|  |  | Number of YES in A4-7, B2, C2, D1, E2 (range 0-8) | … |
